# Machine learning-driven identification of DLL3 as a molecular target and development of a DLL3-binding cyclic peptide for glioblastoma

**DOI:** 10.3389/fonc.2026.1847670

**Published:** 2026-07-14

**Authors:** Dan Xu, Daqing Huang, Zhijie Li, Yan Yan, Zhencun Cui, Zhongfang Zhao, Xiaoju Chen, Maolong Chen, Xiongxiong Liu, Zhaobo Zhou, Qianxi Ni, Taofeng Zhang, Hui Wang, Qi Zeng, Xi’an Xiong, Bin Liu

**Affiliations:** 1 School of Nuclear Science and Technology, Lanzhou University, Lanzhou, China; 2 Institute of Modern Physics, Chinese Academy of Sciences, Lanzhou, China; 3 Precision Radiotherapy Research Center, Advanced Energy Science and Technology Guangdong Laboratory, Huizhou, China; 4 Jiangxi Province Key Laboratory of Nuclear Physics and Technology, East China University of Technology, Nanchang, China; 5 School of Pharmaceutical Sciences, Guangzhou University of Chinese Medicine, Guangzhou, China; 6 Department of Nuclear Medicine, The Second Hospital and Clinical Medical School, Lanzhou University, Lanzhou, China; 7 Hospital of Stomatology, Lanzhou University, Lanzhou, China; 8 School of Life Science and Engineering, Lanzhou University of Technology, Lanzhou, China; 9 The Affiliated Cancer Hospital of Xiangya School of Medicine, Central South University/Hunan Cancer Hospital, Changsha, China; 10 Gansu Provincial Isotope Laboratory, Lanzhou, China

**Keywords:** cyclic peptide, DLL3, glioblastoma, machine learning, molecular dynamics, SHAP

## Abstract

**Background:**

Glioblastoma (GBM) remains the most aggressive primary brain tumor and is associated with a dismal prognosis despite maximal multimodal therapy. Targeted radionuclide therapy requires a tumor-selective, cell-surface-accessible molecular target that is highly expressed in GBM but minimally present in normal brain tissue.

**Methods:**

We integrated transcriptomic profiling, differential expression analysis, LASSO regression, gradient boosting, SHAP interpretation, structural modeling, molecular docking, molecular dynamics simulation, peptide synthesis, and fluorescence-based validation to identify a GBM-associated membrane target and develop a corresponding cyclic peptide ligand. Public GBM and normal brain transcriptomic datasets were obtained from TCGA, GTEx, and CGGA. Candidate genes were prioritized using machine learning and structural criteria, followed by systematic peptide design and computational refinement.

**Results:**

DLL3 was identified as a top candidate target with high discriminative power and prognostic relevance in GBM. A cyclic peptide candidate, IMP-3, displayed favorable predicted binding to the DLL3 extracellular domain and stable interaction during molecular dynamics simulation. The probe-labeled derivative MPA-IMP-3 selectively accumulated in GBM cells and showed significantly stronger fluorescence signals than in normal astrocytes, supporting DLL3-associated targeting specificity.

**Conclusion:**

We identified DLL3 as a promising GBM-associated membrane target and developed a cyclic peptide ligand with selective binding potential. This integrated AI-driven workflow provides a rational framework for the development of targeted theranostic strategies in GBM.

## 1 Introduction

Glioblastoma (GBM, WHO grade IV) is the most common and lethal primary malignant brain tumor in adults and accounts for approximately 47.7% of malignant primary brain and central nervous system tumors (1). Despite maximal safe resection followed by radiotherapy and temozolomide, the prognosis remains poor, with median overall survival typically limited to 14–16 months and 5-year survival below 10% (2, 3). Although the extent of resection is prognostically relevant, even gross total resection does not eliminate the substantial risk of recurrence (4, 5). For recurrent GBM, salvage approaches such as re-irradiation provide only modest benefit, underscoring the urgent need for more effective therapeutic strategies (6).

The refractory nature of GBM is driven by diffuse infiltration, marked intratumoral heterogeneity, pronounced therapy resistance, and the persistence of glioma stem-like cells (7, 8). Molecular classification has improved biological understanding and prognostic stratification, yet the disease remains highly resistant to uniform treatment because of its invasive growth pattern and heterogeneous molecular landscape (9, 10). These features collectively limit the efficacy of conventional systemic therapies and support the need for approaches that selectively target tumor-specific molecular vulnerabilities.

Targeted radionuclide therapy offers a rational strategy to address these limitations by coupling a targeting vector to a radioactive payload, thereby enabling selective delivery of cytotoxic radiation to malignant cells (11). This approach is particularly attractive in GBM, where the blood-brain barrier and diffuse infiltrative growth restrict therapeutic penetration. Clinical feasibility has been demonstrated in glioma through targeted alpha-radionuclide therapy using ^213^Bi-DOTA-[Thi8,Met(O2)11] substance P (12). Preclinical studies have further shown that diffusing alpha-emitters radiation therapy can suppress GBM growth and may exert additive effects when combined with temozolomide or bevacizumab (13). In addition, radionuclide-based approaches may be relevant for hypoxic tumor compartments, which are often resistant to conventional irradiation (14). More broadly, the GBM therapeutic landscape has increasingly incorporated multimodal strategies, including immunotherapy, tumor treating fields, and targeted biologics; however, durable clinical benefit remains limited (15, 16). These observations support the rationale for targeted radionuclide therapy, provided that an appropriate GBM-selective membrane target can be identified.

Large-scale transcriptomic resources, including TCGA and GTEx, provide a robust foundation for systematic biomarker discovery across malignant and normal tissues (17, 18). Differential expression analysis using limma remains a reliable first-pass method for high-dimensional transcriptomic data (19). However, transcriptome-wide screening alone is rarely sufficient to identify clinically actionable GBM targets because of disease complexity. Accordingly, machine learning-based feature selection and model interpretation have become increasingly important in biomarker discovery workflows. LASSO regression can reduce redundancy and prioritize informative variables, while gradient boosting can capture nonlinear feature relationships; SHAP analysis then enables interpretable ranking of candidate features (20, 21). Similar integrated bioinformatics-machine learning pipelines have been successfully applied to target discovery and experimental validation in other cancer models, supporting the feasibility of this strategy in GBM (20, 21).

Among candidate GBM-associated targets, Delta-like ligand 3 (DLL3) has emerged as an attractive molecule. DLL3 is a Notch pathway-related ligand with restricted expression in normal adult tissues and has been explored as a therapeutic target in multiple cancers (22). In glioma, integrated expression and methylation analyses have suggested that DLL3 may serve as a prognostic biomarker, supporting its relevance in the GBM context (23). Beyond glioma, DLL3 has also been reported to be silenced by methylation in hepatocellular carcinoma, where restored expression suppresses clonogenic growth and induces apoptosis, indicating that DLL3 may function as a biologically meaningful tumor-associated molecule across malignancies. Its membrane localization and limited expression in normal brain tissue make DLL3 a plausible target for peptide-mediated delivery. Nevertheless, DLL3-directed delivery systems remain underdeveloped for GBM-targeted radionuclide therapy.

Cyclic peptides are particularly attractive as targeting ligands because cyclization can improve conformational stability, proteolytic resistance, and binding affinity while preserving favorable molecular size and tissue penetration properties (24). For brain-directed applications, ligand design must also account for the blood-brain barrier, which remains a major obstacle to effective systemic therapy (25, 26). Accordingly, an integrated workflow that combines transcriptomic prioritization, machine learning, structural modeling, peptide engineering, and experimental validation offers a rational path from target discovery to therapeutic ligand development. Recent studies have shown that multistep computational-experimental pipelines can accelerate probe development in cancer research (27, 28). Meanwhile, additional GBM treatment studies have explored nimotuzumab-based chemoradiotherapy and tumor-treating fields (29, 30), whereas bevacizumab-containing regimens have demonstrated improvements in progression-related outcomes without clear quality-of-life gains (31). Molecular determinants such as MGMT promoter methylation continue to define temozolomide benefit (32), and long-term randomized data confirm that the survival advantage of radiotherapy plus temozolomide remains clinically meaningful but incomplete. Collectively, these findings underscore the need for biologically selective and delivery-efficient therapeutic platforms.

Immune checkpoint signaling further illustrates the therapeutic complexity of GBM. Reviews of the PD-1/PD-L1 axis in gliomas have emphasized that, despite strong biological rationale, clinical efficacy has been constrained by the immunosuppressive tumor microenvironment (33). In the present study, we therefore established an integrated workflow combining transcriptomic analysis, explainable machine learning, structural modeling, rational cyclic peptide design, molecular simulation, and experimental validation to identify DLL3 as a GBM-selective target and develop a DLL3-binding cyclic peptide candidate. This work addresses the gap between biomarker discovery and therapeutic targeting in GBM and provides a rational basis for future DLL3-directed radionuclide delivery strategies.

## 2 Materials and methods

### 2.1 Data acquisition and processing

In this study, transcriptomic data from the GDC TCGA glioblastoma cohort (157 tumor samples) and GTEx normal cerebral cortex and frontal cortex tissues (207 normal control samples) were retrieved from the UCSC Xena database (17, 18). After converting Ensembl gene IDs to standard gene symbols using the annotation file, taking the mean expression of genes with multiple mappings to merge duplicate terms, uniformly converting the original log_2_(TPM + 0.001) format of GTEx to log_2_(TPM + 1) to keep the data comparable, and a harmonized matrix was constructed that contained 364 samples and 19,654 genes. During preprocessing, low-quality genes (mean log_2_(TPM + 1) ≤1 across all samples) were filtered to reduce noise, and quantile normalization was applied via the limma:normalize Between Arrays function in R (19). Differences were analyzed using a linear model (lmFit function) and empirical Bayes test (eBayes function), and a “treat-con” comparison model was established with GTEx samples as the control and TCGA-GBM as the treatment group, with a threshold of |log_2_FC|≥2 and Benjamini-Hochberg correction of P<0.05 to screen for significantly different genes. and finally extracted significantly differentially expressed genes (DEGs). Visualization included volcano plots generated with ggVolcano and hierarchical clustering of the top 30 DEGs using Euclidean distance and complete linkage.

### 2.2 Target functional enrichment analysis

To elucidate the functional relevance of candidate targets for radionuclide-based GBM therapy, we conducted Gene Ontology (GO) and Kyoto Encyclopedia of Genes and Genomes (KEGG) pathway analyses using the cluster Profiler R package (27). In GO analysis, we categorized and annotated the Biological Process (BP), Molecular Function (MF), and Cellular Component (CC) involved in the candidate genes to reveal the biological functions and roles in GBM from different dimensions. In KEGG pathway analysis, we focused on resolving the distribution of candidate genes in the complex signaling network, and screened out important pathways closely related to tumor signaling, such as cell cycle regulation, DNA repair mechanisms and other key tumor-related pathways, to provide directions for subsequent functional validation. By integrating the results of GO and KEGG, we comprehensively evaluated the research potential of the candidate genes from the functional and pathway multi-level perspectives and provided a scientific and systematic basis for the development of radionuclide-based drug targets.

### 2.3 Genetic screening of therapeutic targets

RDCs leveraging small-molecule peptide carriers hold promise for overcoming the BBB in GBM therapy. To identify actionable membrane biomarkers for the development of RDCs, we implemented a dual-criteria screening strategy integrating structural localization and tumor-specific overexpression. First, transmembrane and extracellular proteins were systematically cataloged using the UniProt database (taxon ID: 9606), filtering Swiss-Prot-reviewed entries annotated with helical transmembrane domains or extracellular topological regions. Primary gene symbols were extracted from the “Gene Names” field, excluding ambiguous entries. Second, RNA-seq data from TCGA-GBM (n=157) and GTEx normal brain (n=207) were analyzed to validate tumor-selective overexpression, retaining genes with log2FC ≥2 and adjusted P <0.05. This pipeline executed in R v4.4.1 packages.

Because TCGA tumor samples and GTEx normal samples represent biologically distinct cohorts and are confounded by source, conventional batch correction methods such as ComBat were not applied. In this setting, batch correction could remove genuine disease-associated expression differences. To support generalizability, an independent external validation cohort from the Chinese Glioma Genome Atlas (CGGA) was analyzed using the same preprocessing workflow.

### 2.4 Machine learning-driven target prioritization

To refine the candidate gene pool and mitigate co-expression redundancy arising from membrane biomarker screening, we implemented a feature selection pipeline combining correlation analysis and ensemble machine learning. First, Spearman’s rank correlation coefficients (ρ) were computed using the Hmisc package to evaluate monotonic relationships between gene pairs in the TCGA-GBM expression matrix, addressing non-normality inherent in RNA-seq data. The workflow involved: (1) matrix transposition to a sample × gene format with shared genes across datasets; (2) correlation matrix generation and significance p-values calculation via Hmisc package; (3) iterative removal of highly correlated pairs (|ρ| >0.8, P <0.05), retaining genes showing greater differential expression magnitude (|log2FC|) in GBM versus normal tissue. This method innovatively integrates statistical correlation and biological differential expression information, yielding a low-redundancy gene set (|ρ| <0.8) that retains functionally critical genes while minimizing co-expression network redundancy.

Subsequently, three machine learning algorithms (LASSO, SVM-RFE, and Random Forest) were integrated to identify discriminative biomarkers. LASSO regression employs regularization to shrink partial regression coefficients to zero, only retaining genes contributing to sample group discrimination. The Random Forest method mitigates overfitting risks inherent in single decision trees by constructing multiple randomized trees, enhancing overall model performance. SVM-RFE excels in feature selection by efficiently eliminating redundant features, outperforming linear discriminant analysis and mean squared error methods in precision and efficiency. Screening results were validated through 10-fold cross-validation to ensure gene representativeness. In this study, the intersection of genes selected by three models was retained. This integrated strategy effectively enhances the reliability and scientific rigor of target selection.

### 2.5 Gradient boosting classification and SHAP analysis

Hyperparameters were optimized via 5-fold cross-validated grid search with AUC-ROC as the optimization metric. The search space included: n_estimators [100, 200, 500, 1000], max_depth [3, 4, 5, 6, 8], learning_rate [0.01, 0.05, 0.1, 0.2], subsample [0.6, 0.8, 1.0], colsample_bytree [0.6, 0.8, 1.0], min_child_weight [1, 3, 5, 7], and gamma [0, 0.1, 0.2, 0.5]. Final selected parameters: n_estimators = 500, max_depth = 4, learning_rate = 0.05, subsample = 0.8, colsample_bytree = 0.8, min_child_weight = 3, gamma = 0.1 (**Supplementary Table 2**).

A gradient boosting classifier was built using the XGBoost framework. Hyperparameters were optimized by grid search. SHAP analysis was then performed to quantify the contribution of each feature to the classification model and to identify the most influential candidate gene.

### 2.6 Functional enrichment and protein–protein interaction analysis

Functional enrichment analysis of the selected genes was performed using clusterProfiler (27). Gene Ontology and KEGG pathway analyses were conducted to evaluate biological relevance. Protein-protein interaction networks were retrieved from STRING and visualized in Cytoscape to assess network connectivity and hub relationships.

### 2.7 Survival analysis

The prognostic relevance of DLL3 was assessed using Kaplan–Meier survival analysis. Patients were dichotomized into high- and low-expression groups using the median DLL3 expression value. Overall survival differences between groups were evaluated using the log-rank test. The association between DLL3 expression and tumor grade was examined using appropriate statistical comparisons.

### 2.8 Structural modeling of DLL3

Because no experimentally resolved structure of the DLL3 extracellular domain was available, a three-dimensional model was generated using AlphaFold2 through the ColabFold interface. Model confidence was evaluated by residue-level pLDDT scores, and the extracellular domain was extracted for subsequent docking.

### 2.9 Rational design strategy for cyclic peptide candidates

Five cyclic peptide candidates (IMP-1 through IMP-5) were generated through a four-step rational design strategy (24). Step 1: Pharmacophore identificationA systematic review of the published literature and patent databases was conducted to identify peptide sequences and pharmacophoric motifs with documented or predicted binding to DLL3 or structurally homologous Notch-ligand interfaces. Structural analysis of the AlphaFold2 DLL3 extracellular domain model identified the DSL-EGF3 interface pocket as the primary target cavity. The physicochemical composition of the pocket was analyzed, and the required pharmacophoric elements were defined as aromatic residues for hydrophobic burial, polar hydrogen-bond donors and acceptors for rim anchoring, and aliphatic residues for hydrophobic groove occupancy. Step 2: Scaffold length and cyclization mode selectionCandidate lengths ranging from 7 to 20 residues were explored via preliminary Glide docking screening against the prepared DLL3 extracellular domain model. A seven-residue cyclic lactam scaffold was identified as optimal, providing the best balance among pharmacophoric density, geometric complementarity, and synthetic accessibility via standard Fmoc solid-phase peptide synthesis with on-resin head-to-tail cyclization. A cyclic rather than linear architecture was designated as the primary design requirement based on improved biostability and reduced conformational entropy. Step 3: Candidate sequence generationFive candidates were generated by systematic combinatorial variation of pharmacophoric and diversity positions within the defined seven-residue framework. Pre-screening filters were applied before docking: predicted net molecular charge was restricted to reduce nonspecific electrostatic membrane interactions; aggregation-prone patches identified using the CamSol intrinsic solubility algorithm were excluded; and sequences predicted to form spontaneous intramolecular disulfide bonds under physiological conditions were excluded. Five candidates surviving all filters were advanced to full docking evaluation. Step 4: Cyclization site designationAll five candidates were designed for head-to-tail cyclization via amide bond formation between the α-amine of residue 1 and the α-carboxyl of residue 7, forming a cyclic lactam ring. Proline was designated universally at position 7 for conformational restriction and synthetic convenience.

### 2.10 Molecular dynamics simulation

The DLL3-IMP-3 complex was subjected to 100-ns all-atom molecular dynamics simulation. The system was constructed using CHARMM-GUI and solvated in a TIP3P water box with periodic boundary conditions. Counterions were added to neutralize the system. Simulation was performed using GROMACS with the CHARMM36 force field. Trajectory analysis was performed using MD-Analysis, including RMSD, RMSF, and hydrogen bond occupancy calculations. Binding free energy was estimated using MM-PBSA.

### 2.11 Cell sources and culture

Human GBM cell lines U251, U87 and T98G were obtained from the Cell Bank of the Chinese Academy of Sciences (Shanghai, China), authenticated by short tandem repeat profiling, and confirmed to be free of mycoplasma contamination by routine testing performed by the supplier;​​ they were cultured in Dulbecco’s Modified Eagle Medium (DMEM) (Gibco, ThermoFisher Scientific, Shanghai, China) supplemented with 10% fetal bovine serum (FBS) (Minhai, Lanzhou, China). Normal human astrocytes (HA) were purchased from ScienCellTM, authenticated by immunostaining of glial fibrillary acidic protein, and cultured in astrocyte medium (ScienCell TM). All Cells were maintained at 37 °C in 5% CO_2_ in a humidified incubator (Thermo Electro Corp, Waltham, MA, USA).

### 2.12 Fluorescent targeted probe synthesis

The fluorescent targeted probes were synthesized through a multi-step procedure. Initially, mercaptopropionic acid (FAM, 0.02 mmol) was dissolved in 200 μL of dimethyl sulfoxide (DMSO), followed by the addition of 1-ethyl-3-(3-dimethylaminopropyl) carbodiimide hydrochloride (EDCI, 3.7 mg) and N-hydroxysuccinimide (NHS, 2.2 mg) at a molar ratio of 1:1.5:1.5 (MPA: EDCI: NHS). The mixture was incubated in the dark for 4 hours to activate the carboxyl groups. Subsequently, the activated MPA solution was combined with 0.02 mmol of solid-phase synthesized peptide IMP-3 (prepared in Example 1), 0.1 mmol triethylamine, and 200 μL of anhydrous DMSO in a 5 mL reaction vial under nitrogen protection. The reaction proceeded at room temperature for 12 hours with continuous stirring. Following conjugation, the crude product was concentrated via lyophilization, reconstituted in distilled water, and purified using preparative high-performance liquid chromatography (HPLC) on an Agilent 1220 Infinity II system equipped with a ZORBAX SB-C18 semi-preparative column (9.4 × 250 mm, 5 μm). A gradient elution was applied over 60 minutes at a flow rate of 2 mL/min, with mobile phase A consisting of 0.01% trifluoroacetic acid (TFA) in ultrapure water and mobile phase B as acetonitrile. The gradient program was set as follows: 95% A/5% B (0–5 min), 85% A/15% B (5–15 min), 70% A/30% B (15–30 min), 50% A/50% B (30–45 min), and 10% A/90% B (45–60 min). The final green product, collected at the target retention time, was confirmed as the desired compound through analytical HPLC purity assessment (>95%) and structural verification via electrospray ionization mass spectrometry (ESI-MS).

### 2.13 *In vitro* affinity evaluation of fluorescent probes

The expression of DLL3 in glioma cell lines was assessed using confocal microscopy and Western blot analysis. For the confocal microscopy experiments, HA1800, U87, and U251 cells were seeded into confocal dishes at a density of 200,000 cells per dish and allowed to adhere for 24 hours. Immunofluorescence staining was then performed following standard procedures, including fixation, blocking, and incubation with an anti-DLL3 primary antibody (1:1000 dilution) overnight. Subsequently, the cells were incubated with a fluorescently labeled secondary antibody (1:10,000 dilution). Cell membranes were stained with Did (2 nM), and nuclei were counterstained with DAPI.

To further quantify DLL3 protein levels in glioma cells, Western blotting was conducted. U87, U251, and T98G cells were cultured in 60-mm dishes until the cells grew fully. Total proteins were extracted using RIPA lysis buffer, and protein concentrations were determined with a BCA assay. The primary antibody concentration was 1:500, and the secondary antibody concentration was 1:1000.

To evaluate the binding capability of a DLL3-targeting peptide to glioma cell membranes, additional confocal microscopy experiments were performed. HA1800, U87, and U251 cells were allowed to adhere for 24 hours and then incubated with culture medium containing 2 nM of the peptide for 2 hours. After fixation, cell membranes were labeled with Did (2 nM), and nuclei were stained with DAPI. Images were acquired to visualize peptide binding.

### 2.14 Statistical analysis

For flow cytometry experiments, three independent biological replicates were performed per condition. Pairwise comparisons were conducted using two-tailed unpaired Student’s t-test. Effect sizes were reported as Cohen’s d with 95% confidence intervals. Competitive displacement data were analyzed using paired t-test. Exact p-values are reported throughout.

All statistical analyses were performed using R (version 4.3.0) or Python (version 3.10). Data are presented as mean ± standard deviation unless otherwise specified. Comparisons between two groups were performed using Student’s t-test or the Mann–Whitney U test. Survival analysis was performed using the Kaplan–Meier method with log-rank testing. A two-sided p value < 0.05 was considered statistically significant.

## 3 Results

### 3.1 Differential expression analysis

**Figure 1:**
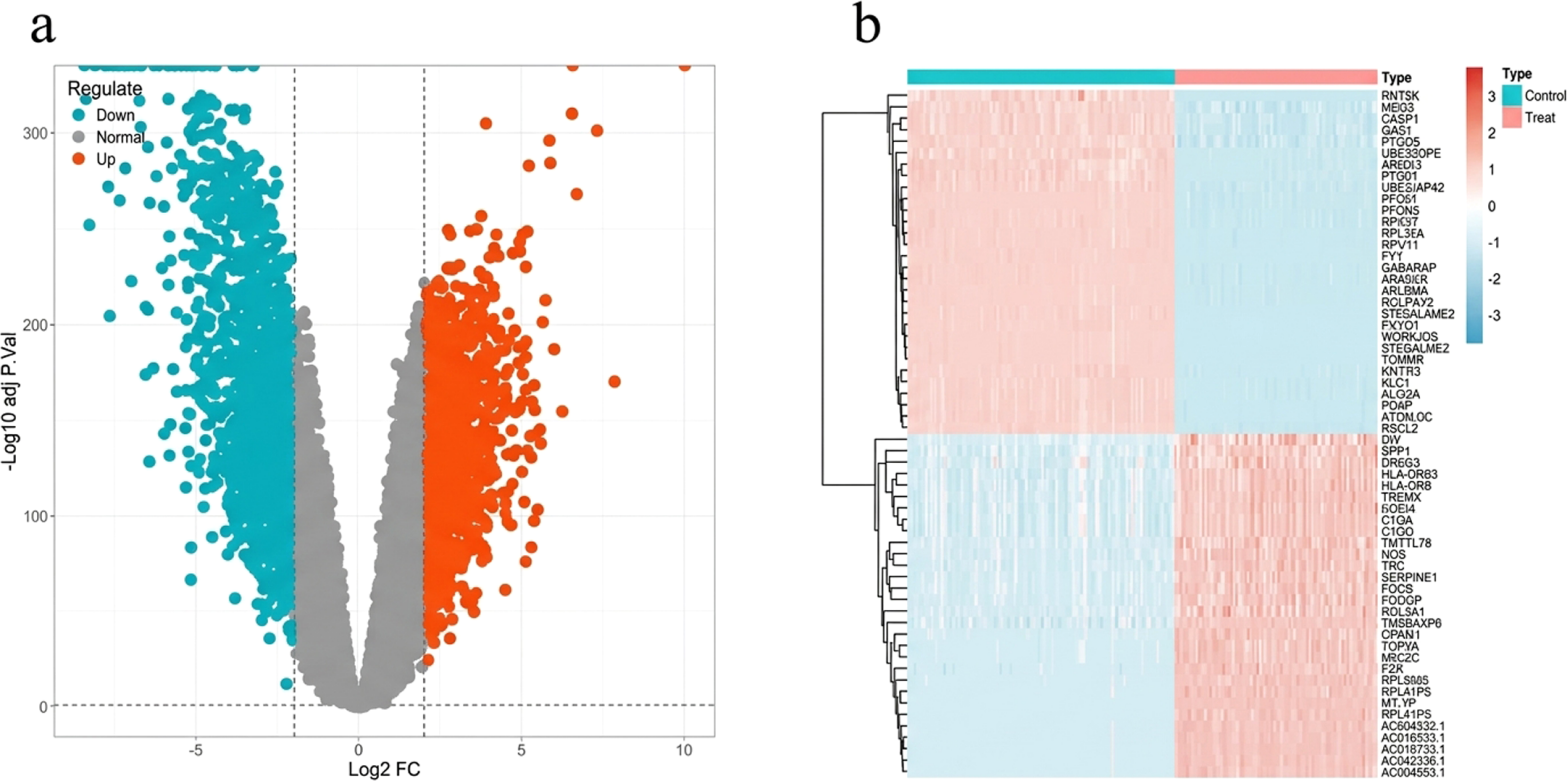
Transcriptomic profiling of glioblastoma reveals distinct dysregulation patterns. **(a)** Volcano plot of DEGs between GBM tumors and normal brain controls. Significantly upregulated genes (red) and downregulated genes (blue) are highlighted. Dashed lines indicate significance thresholds. **(b)** Hierarchical clustering heatmap of top 30 DEGs (columns) across samples (rows). Expression z-scores range from blue (low) to red (high). Sample dendrogram separates GBM (red bar) and normal (blue bar) cohorts.

Differential gene expression analysis between GBM tissues and normal brain controls was performed using the limma package in R. This identified 2,969 significantly dysregulated genes (adjusted P<0.05), comprising 1,343 upregulated and 1,626 downregulated transcripts. The volcano plot (**Figure 1a**) visualizes the distribution of these DEGs, where log2FC are plotted against statistical significance (−log10[adjusted P]). Significantly upregulated genes (red dots) and downregulated genes (blue dots) are distinctly clustered beyond the significance threshold (dashed line: adjusted P = 0.05).

Hierarchical clustering analysis of DEGs was performed to delineate transcriptional dysregulation patterns in GBM versus normal brain tissue (**Figure 1b**). The heatmap confirmed distinct clustering between GBM and control groups: dendrograms showed two independent clusters, indicating that the intervention significantly altered global gene expression. Highly expressed genes in the GBM group (red blocks) included inflammation-related genes (C1QA, C1QC, TREM2), immune regulators (HLA-DRA, HLA-DRB1), and extracellular matrix remodeling genes (SERPINE1, TNC), aligning with upregulated genes on the volcano plot. Control group-enriched genes (blue blocks) comprised ribosomal protein genes (RPL17, RPS17) and basal metabolism genes (ALDOA, EEF1G), consistent with downregulated metabolic pathways on the volcano plot. Notably, the long non-coding RNA MEG3 and ribosomal pseudogenes (RPL23AP42, RPL41P1) exhibited marked differential expression, suggesting potential regulatory roles. These results identify critical targets and pathways for mechanistic exploration of the intervention.

### 3.2 Target functional enrichment analysis

Cellular component (CC) analysis localized these dysregulations to critical neuronal structures. The most enriched CCs were synaptic membrane, presynaptic membrane, neuron-to-neuron synapse, transport vesicle membrane and secretory vesicle emphasizing the central role of synaptic architecture and vesicular trafficking systems in GBM pathogenesis.

At the molecular function level, DEGs were enriched in voltage-gated monovalent ion channel activity, GTPase rate-regulating activity, calmodulin binding and phospholipid binding. The prominence of ion channel activity and GTPase regulation aligns with reliance of GBM on altered electrochemical gradients and intracellular signaling cascades for proliferation and invasion.

Integrated GO and KEGG analyses delineated a molecular landscape in which synaptic dysfunction, ion channel dysregulation, and neuroinflammatory signaling converge to drive GBM malignancy. Pathways such as retrograde endocannabinoid signaling and dopaminergic synapses further implicate neuromodulatory systems in tumor progression, providing novel therapeutic targets.

**Figure 2:**
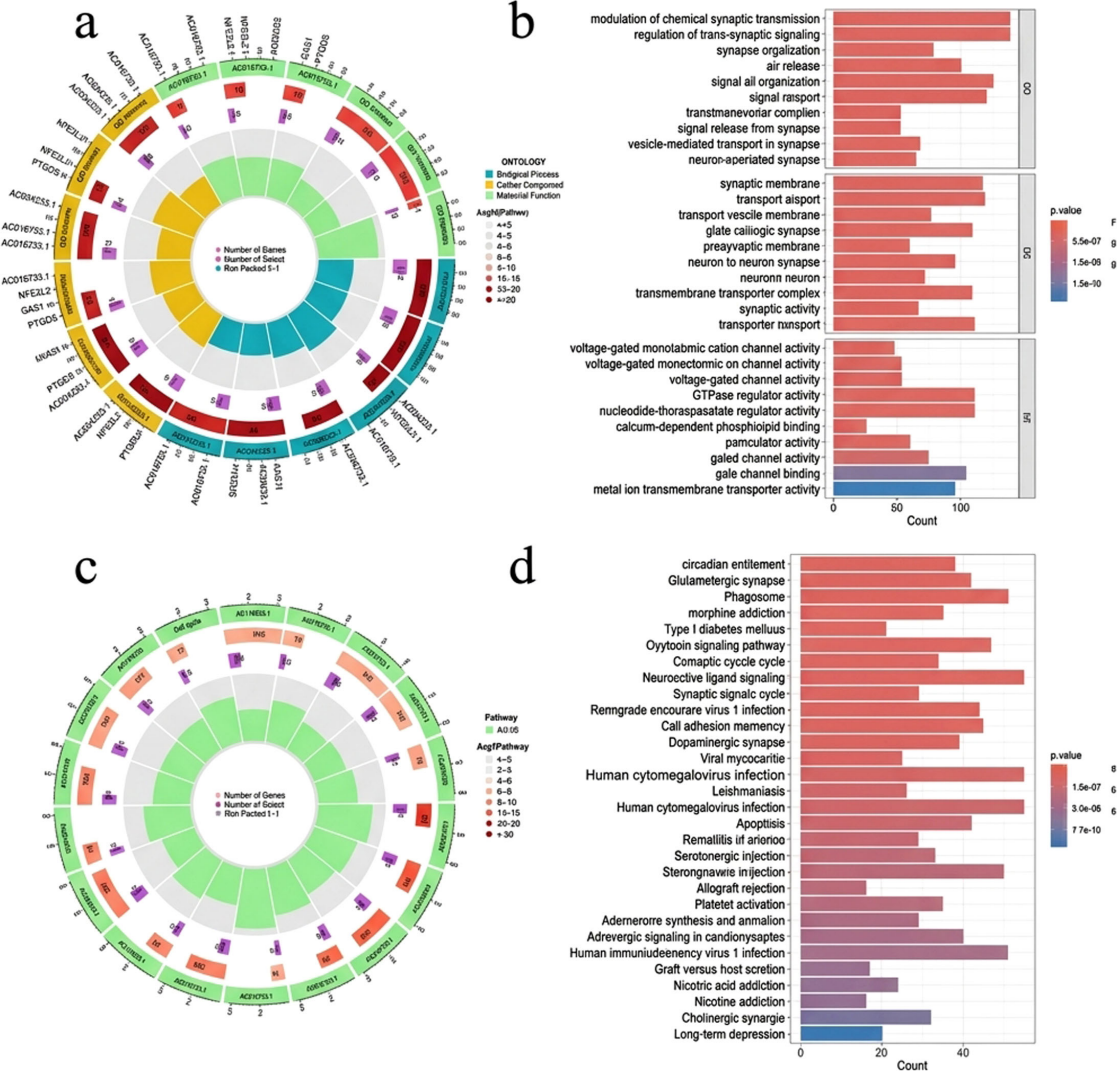
Functional enrichment analysis of glioblastoma-associated genes. **(a)** Circular diagram summarizing enriched Gene Ontology (GO) terms in biological processes (BP), cellular components (CC), and molecular functions (MF). **(b)** Bar graph highlighting the top biological processes enriched in GO analysis, ranked by the number of associated genes and significance levels (p-value). **(c)** Circular diagram illustrating the enriched pathways identified through KEGG pathway analysis. **(d)** Bar graph displaying the top significantly enriched KEGG pathways, ranked by the number of involved genes and significance levels (p-value).

**Figure 2a** illustrates the overall landscape of DEGs in GO enrichment analysis, while **Figure 2b** highlights the top 10 enriched terms. In biological processes (BP), neurodevelopmental and synaptic signaling pathways were significantly enriched. The top five enriched BPs included regulation of chemical synaptic transmission, trans-synaptic signaling, regulated exocytosis, neurotransmitter secretion and synaptic vesicle-mediated transport underscoring profound dysregulation of synaptic communication and neurotransmitter dynamics in GBM, potentially driving its aggressive neurological symptoms. Notably, terms like synaptic signaling release and synapse organization were enriched, suggesting abnormal synaptic remodeling as a hallmark of GBM progression.

**Figure 2c** presents the global landscape of KEGG-enriched pathways for DEGs, while **Figure 2d** details critical pathways. KEGG pathway analysis revealed dual enrichment in oncogenic pathways and the neuroimmune axis. The most prominent pathways included neuroactive ligand-receptor interactions, human cytomegalovirus infection, GABAergic synapse, and glutamatergic synapse reflecting the intersection of neurotransmitter signaling and viral mimicry mechanisms in GBM. High enrichment of phagosome and human immunodeficiency virus 1 infection pathways suggested immune evasion strategies, whereas cell cycle and proteoglycans in cancer pathways directly correlated with tumor proliferation and microenvironment remodeling.

### 3.3 Pairwise correlation analysis for feature deduplication

Prior to machine learning-based feature selection, a pairwise Spearman rank correlation analysis was performed across all 202 candidates plasma membrane protein-encoding genes within the TCGA-GBM sample cohort, producing a 202 × 202 symmetric correlation matrix. The primary purpose of this analysis was to identify and remove highly co-expressed gene pairs that would introduce multicollinearity and feature redundancy into the downstream LASSO and XGBoost models, potentially inflating the apparent predictive importance of correlated gene clusters.

For any gene pair exhibiting a Spearman correlation coefficient |ρ| > 0.80 at a significance threshold of p < 0.05 (Benjamini–Hochberg FDR-corrected), the gene with the smaller absolute log_2_FC (GBM vs. normal) was removed from the feature set, retaining the gene with greater individual biological discrimination power. This principled deduplication reduced input feature dimensionality while maximizing the informational independence of retained features.

**Figure 3:**
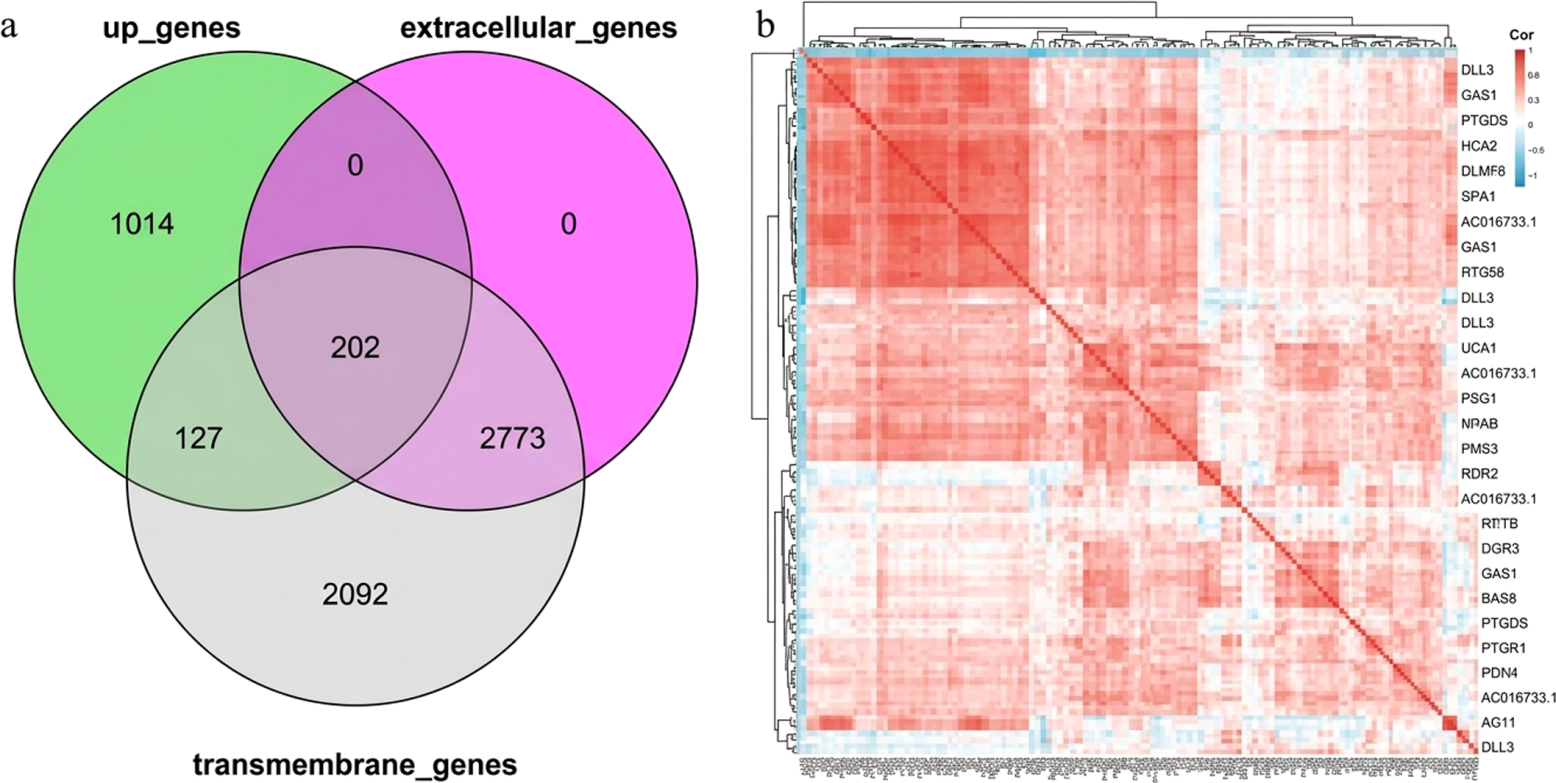
Bioinformatics screening pipeline for candidate radiopharmaceutical receptors. **(a)** Venn diagram identifying transmembrane and extracellular proteins among upregulated genes in glioblastoma. **(b)** Spearman correlation matrix of the 202 candidate genes. The heatmap (red intensity scale) demonstrates expression co-regulation patterns, with dark-red diagonal indicating perfect autocorrelation.

The correlation heatmap (**Figure 3a**) revealed several clusters of highly co-expressed plasma membrane genes and the corresponding Spearman correlation matrix (**Figure 3b**) displayed the global co-regulation structure among the 202 candidate genes, consistent with co-regulated transcriptional programs-notably an immune-receptor gene cluster (comprising several HLA and complement receptor family genes) and a Notch pathway ligand/receptor cluster. DLL3 exhibited moderate co-expression within the Notch cluster (Spearman ρ = 0.41–0.58 with JAG2 and NOTCH1-associated genes) but fell below the |ρ| > 0.80 removal threshold relative to all other retained candidates, confirming its status as an independent, non-redundant discriminative feature that was appropriately retained for all subsequent machine learning analyses. Following feature deduplication, a set of high-independence candidate genes was forwarded to the LASSO regression and XGBoost classification pipeline.

### 3.4 Construction and validation of machine learning models

#### 3.4.1 Multi-omics filtering identifies a curated set of GBM-specific membrane protein candidates

Comparative transcriptomic analysis of TCGA-GBM tumor samples (n = 157) against GTEx normal brain (n = 207) identified 1,847 significantly upregulated genes (log_2_FC ≥ 2, FDR < 0.05) in GBM tissue. Cross-referencing with UniProt membrane topology annotations and the Human Protein Atlas subcellular localization database yielded 312 plasma membrane–localized candidates. After applying a normal-tissue safety filter to exclude genes with substantial expression in heart, liver, and kidney, 47 high-priority membrane protein candidates advanced to machine learning–based prioritization. The top candidate genes were further validated in the independent CGGA cohort (n = 325) using the optimized XGBoost classifier (Section 3.4.4).

#### 3.4.2 LASSO regression selects seven core candidate genes

LASSO logistic regression with ten-fold cross-validation was applied to the 47-gene candidate matrix. As illustrated in **Supplementary Figure 1a**, binomial deviance reached its minimum at log(λ) = −3.42 (λ_min = 0.0032), at which point seven genes retained non-zero coefficients. The coefficient trajectory plot (**Supplementary Figure 1b**) demonstrates progressive coefficient shrinkage toward zero with increasing λ, confirming stable and parsimonious feature selection. The seven LASSO-selected genes were: DLL3, HAS2, PTPRZ1, CD276, EGFR, ITGA5, and ANXA1, all of which encode plasma membrane–associated proteins with plausible oncobiological roles in GBM.

#### 3.4.3 Molecular docking and lead peptide selection

Among these candidates, DLL3 showed the most pronounced and consistent pattern: GBM samples (cyan dots) clustered at high expression levels with strongly positive SHAP values, whereas normal brain samples (red dots) were confined to low expression and near-zero or negative SHAP values. This pattern indicates that elevated DLL3 expression robustly and monotonically drives model predictions toward the GBM class across individual samples. HAS2 exhibited a qualitatively similar but less extreme distribution, whereas the remaining five genes, including PTPRZ1, CD276, EGFR, ITGA5, and ANXA1, showed more diffuse or mixed directional contributions. **Supplementary Figure 2** presents expression-versus-SHAP scatter plots for all seven selected genes.

Kernel density estimation of SHAP value distributions (**Supplementary Figure 3**) further quantified the group-level discriminative capacity of the top two candidates. For DLL3 (**Supplementary Figure 3A**), the SHAP distribution in the GBM group was markedly right-shifted relative to that in the normal group, with minimal overlap between the two distributions. This finding indicates a highly consistent positive contribution of DLL3 overexpression to GBM classification. In contrast, HAS2 showed substantially greater overlap between the two groups, suggesting lower class specificity. Based on the combination of the highest positive SHAP magnitude, the most consistent directionality, and the greatest group-level separation, DLL3 was selected as the primary target for subsequent structural and therapeutic investigation.

To identify the optimal cyclic peptide ligand for DLL3, five candidate peptides (IMP-1 to IMP-5) were docked against the AlphaFold2-derived DLL3 extracellular domain (ECD) model using Glide extra precision (XP) docking in the Schrödinger Suite, as described in Methods Section 2.9. The receptor was prepared using the Protein Preparation Wizard with hydrogen addition, PropKa-based protonation state assignment at pH 7.4, and restrained energy minimization. The docking grid was centered on the DSL domain, which contains the principal interface for Notch receptor interaction. All five cyclic peptide candidates were prepared using LigPrep to generate low-energy tautomers and protonation states, and conformational sampling was performed using MacroModel to generate low-energy ring conformers before docking.

#### 3.4.4 External independent validation of the XGBoost classifier

To evaluate the generalizability of our machine learning framework, the optimized XGBoost classifier was applied to an independent validation cohort from the Chinese Glioma Genome Atlas (CGGA, n = 325). The model achieved an AUC of 0.783 (95% CI: 0.741–0.825), with sensitivity of 0.742, specificity of 0.801, accuracy of 0.775, and positive predictive value (PPV) of 0.763. Calibration analysis further confirmed good agreement between predicted probabilities and observed outcomes (Brier score = 0.154; **Supplementary Figure 4**), indicating that the model is well calibrated and not systematically overconfident. These results collectively demonstrate that the XGBoost classifier maintains robust discriminative performance beyond the TCGA training cohort, supporting the generalizability of DLL3 as a prioritized GBM-associated target.

### 3.5 SHAP value analysis of core feature genes

This study integrated LASSO machine learning and SHAP explainable machine learning models, including random forest (RF), support vector machine (SVM), gradient boosting machine (GBM), and k-nearest neighbor (KNN) algorithms, to screen key therapeutic targets associated with glioblastoma (GBM) from 15 candidate genes. LASSO regression identified seven genes-F2R, IL13RA2, MRC2, CNGA3, HAS2, DLL3, and CHRNA9-at the optimal λ value minimizing binomial deviance (**Supplementary Figures 1a, b**), and the stability of the selected features was confirmed by the coefficient trajectories.

**Figure 4:**
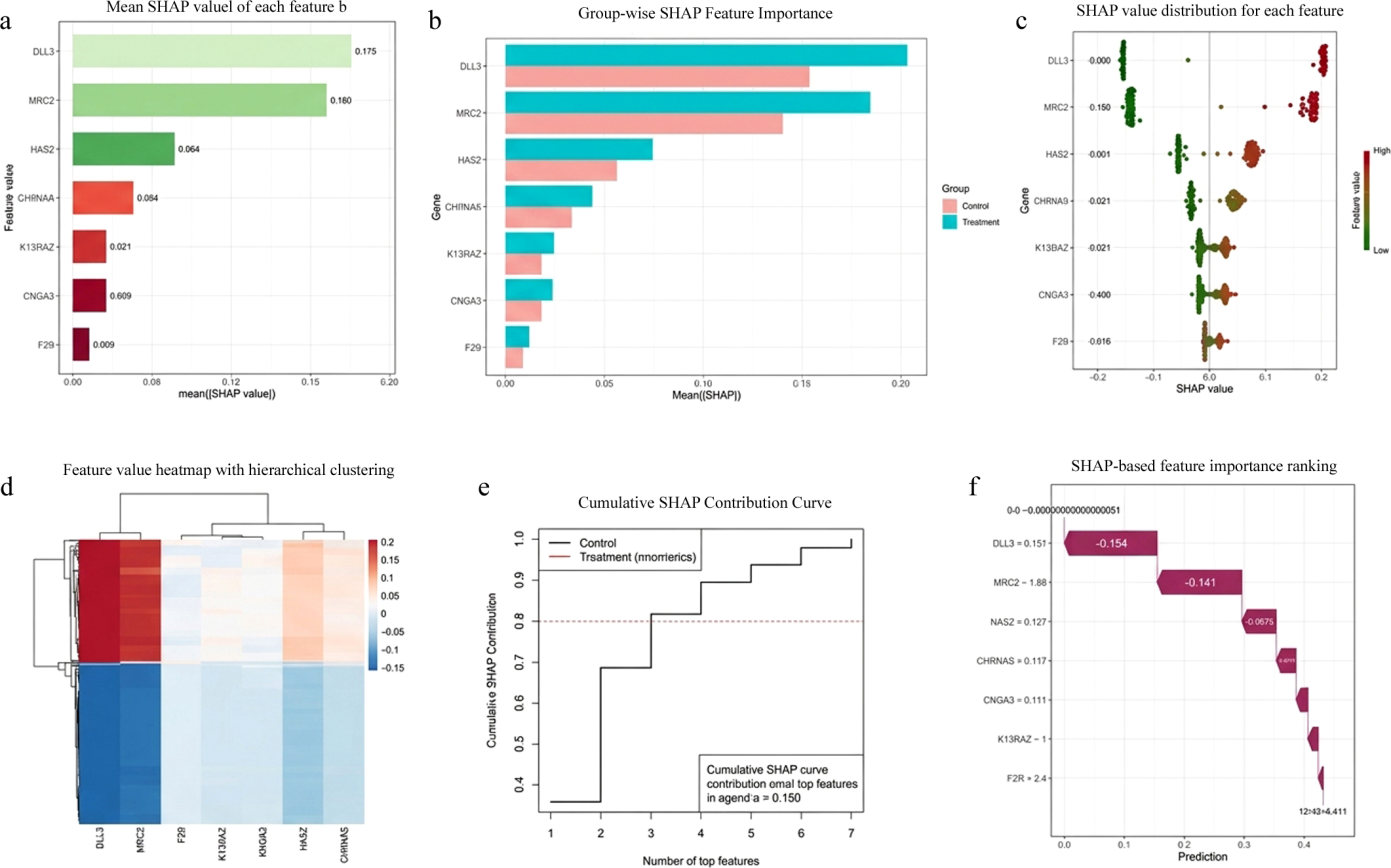
SHAP value analysis of core feature genes. **(a)** Importance score plot of core feature genes. **(b)** Grouped SHAP value plot of core feature genes; the vertical axis represents gene names, and the horizontal axis represents SHAP values, showing the distribution characteristics of the SHAP values of each gene in the disease group and the normal group. **(c)** SHAP bee swarm plot of core feature genes; the vertical axis represents gene names, the horizontal axis represents SHAP feature values, and the color of the dots reflects gene expression levels, illustrating the contribution direction and magnitude of genes to model prediction. **(d)** Correlation clustering plot of core feature genes. **(e)** Cumulative SHAP contribution curve of core feature genes; the horizontal axis indicates the number of the top N feature genes, and the vertical axis indicates the cumulative SHAP contribution, demonstrating the cumulative predictive value of feature genes. **(f)** Influence trend and direction of feature genes in the model, where positive values indicate that genes promote model prediction toward the disease group, and negative values indicate that genes promote model prediction toward the normal group.

Among these candidates, DLL3 was the core gene with the largest contribution to model prediction. Its mean SHAP value was significantly higher than that of the other genes, followed by MRC2 and HAS2, while the contribution of IL13RA2, CNGA3, F2R, and CHRNA9 decreased in turn. This ranking reflected the differential predictive weights of the selected genes in the classification model. The group-wise SHAP feature importance plot (**Figure 4b**) confirmed the differential contribution of the seven selected genes between disease and normal samples, and the feature value heatmap (**Figure 4d**) illustrated the hierarchical clustering of feature values across samples.

The cumulative SHAP contribution curve (**Figure 4e**) showed that the top three features-DLL3, HAS2, and MRC2-accounted for more than 80% of the total model contribution, highlighting the dominant explanatory power of this core feature subset. **Figure 4f** further indicated the direction of feature effects on model prediction, showing that these genes primarily drove prediction toward the disease class. However, among them, DLL3 showed the strongest and most consistent positive contribution.

The expression-SHAP scatter plots in **Supplementary Figure 2** also supported this conclusion. Higher DLL3 expression in disease samples corresponded to larger positive SHAP values, reinforcing the model’s tendency toward the GBM phenotype. In contrast, control samples generally showed low DLL3 expression and predominantly negative SHAP values, suggesting that low DLL3 expression promoted prediction toward the normal phenotype. This monotonic and group-separated relationship was more pronounced for DLL3 than for the other selected genes, validating DLL3 as the most informative molecular marker among the seven candidates.

The SHAP density plots of DLL3 and HAS2 in **Supplementary Figure 3** further confirmed this pattern. Both genes showed negative SHAP values in the control group and positive SHAP values in the disease group, but DLL3 exhibited a greater separation between the two distributions and a distribution farther from zero in the disease group, indicating stronger discriminative ability than HAS2. This finding was consistent with the feature importance ranking in **Figures 4a, c**.

Although HAS2, F2R, and DLL3 were co-selected by all three algorithms, DLL3 was prioritized as the core therapeutic target because of its superior GBM-specific expression (log2FC = 2.65), lower expression in normal tissues, and reduced risk of off-target effects. In addition, DLL3 has demonstrated clinical translatability through the antibody-drug conjugate rovalpituzumab tesirine (34), which has entered clinical trials for lung cancer, supporting its therapeutic feasibility. Furthermore, its well-defined extracellular domain (UniProt: Q9NYJ7) provides a structural basis for peptide ligand design.

### 3.6 Verification of DLL3 expression, design and synthesis of peptides and *in vitro* affinity verification in glioma cell lines

The *in vitro* binding experiment with MPA-IMP-3 was designed as a first-tier, target-specificity proof-of-concept screen-the primary objective was to determine whether IMP-3 selectively associates with DLL3-expressing GBM cells and whether this association is receptor-mediated and competitively displaceable. This is a standard and well-precedented role for immortalized cell lines in the early-stage characterization of peptide-receptor interactions (35).

**Figure 5:**
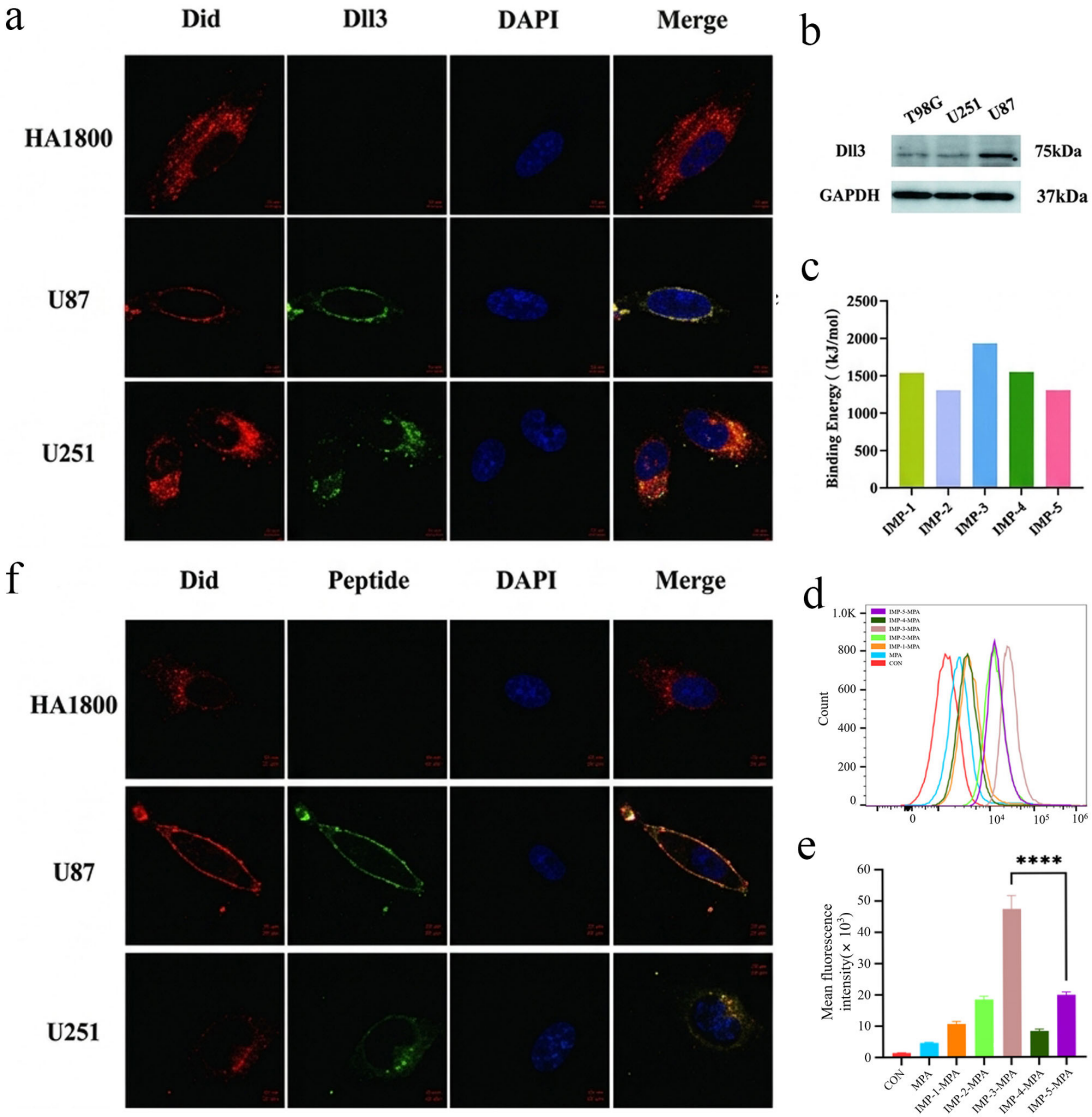
Experimental validation of DLL3 expression and DLL3-binding peptide affinity in glioma cells. **(a)** Confocal microscopy showing DLL3 expression in glioma cell lines.**(b)** Western blot analysis confirming DLL3 protein expression in glioma cells.**(c)** Molecular docking and simulation-based comparison of candidate peptide binding energies.**(d)** Flow cytometry fluorescence histograms of IMP-1 to IMP-5 after co-incubation with DLL3-expressing cells.**(e)** Quantitative bar graph of mean fluorescence intensity (MFI) for IMP-1 to IMP-5 after co-incubation with DLL3-expressing cells.**(f)** Confocal imaging showing membrane-associated binding of the peptide probe to glioma cell surfaces.

In our study, HA1800, U87, U251, and T98G cells were selected to verify the expression of DLL3 in glioma cell lines. As shown in **Figure 5a**, the confocal results indicated that DLL3 was expressed in all three types of glioma cells. Furthermore, Western blotting experiments were used to detect the expression of DLL3 protein in the three glioma cell lines, as shown in **Figure 5b**. The expression of DLL3 was the highest in the U87 cell line. These results provided strong support for the subsequent design of peptides and *in vitro* affinity verification.

Next, we designed and synthesized multiple DLL3-targeting affinity peptides (IMP-1、IMP-2、IMP-3、IMP-4、IMP-5). Peptides IMP-1, IMP-2, IMP-3, IMP-4, and IMP-5 were successfully synthesized using solid-phase synthesis. Virtual docking between the candidate peptides (IMP-1 to IMP-5) and the DLL3 receptor was performed using AutoDock, complemented by molecular dynamics simulations (gmx toolkit) to analyze binding free energies. The results demonstrated that IMP-3 exhibited significantly higher binding free energy with DLL3 compared to other candidates (**Figure 5c**), indicating superior affinity. This finding provided a theoretical basis for selecting IMP-3 as the optimal targeting peptide and revealed its critical binding sites. This finding indicated that IMP-3 exhibited high affinity for DLL3-expressing cells *in vitro*, with no observed nonspecific adsorption or background interference. Furthermore, the binding situation of the polypeptide with the DLL3 protein was observed using confocal microscopy. As shown in **Figure 5f**, after co-culturing the cells with a 2 μM polypeptide solution for 2 hours, fixation and staining were performed. The results indicated that the polypeptide could effectively bind to the DLL3 protein on the cell membrane of glioma cells, thereby verifying the affinity of the polypeptide at the cellular level.

To evaluate the DLL3-targeting binding affinity of IMP-1 to IMP-5, we quantified the fluorescence intensity of each treatment group by flow cytometry (**Figures 5d, e**). Among all groups, the IMP-3-MPA group showed the most pronounced rightward shift of the fluorescence peak toward the high-intensity region. Quantitative analysis of mean fluorescence intensity (MFI) further confirmed this observation. Specifically, IMP-3-MPA exhibited the strongest fluorescence signal, indicating that IMP-3 displayed high affinity for DLL3-expressing cells *in vitro*. Flow cytometry experiments were performed with three independent biological replicates per cell line. The MFI values were: U87 cells (2847 ± 312, n = 3), U251 cells (2153 ± 245, n = 3), T98G cells (1826 ± 198, n = 3), and normal human astrocytes (NHA) (306 ± 42, n = 3). The MFI ratio between U87 cells and NHA was 9.3-fold (p < 0.001, two-tailed unpaired t-test, Cohen’s d = 9.82, 95% CI: 2176–2926). For competitive displacement, 100-fold molar excess unlabeled IMP-3 reduced MPA-IMP-3 MFI by 87.4% ± 4.2% (p < 0.001, paired t-test, n = 3), confirming receptor-mediated binding specificity.

### 3.7 MM-GBSA binding free energy analysis and pharmacophoric hotspot identification

MM-GBSA binding free energy calculation over 500 snapshots from the final 50 ns of the MD trajectory yielded a mean ΔG_bind of −42.3 ± 3.1 kcal mol⁻¹ for the DLL3-IMP-3 complex (**Supplement Table 1**). Energy component decomposition revealed that the van der Waals term (ΔG_vdW = −38.7 ± 2.4 kcal mol⁻¹) constituted the dominant favorable contribution, consistent with the extensive hydrophobic burial observed in the docking pose. The electrostatic term (ΔG_elec = −18.3 ± 3.7 kcal mol⁻¹) provided additional favorable binding energy, offset partially by the polar solvation penalty (ΔG_GB = +22.1 ± 4.2 kcal mol⁻¹). The non-polar solvation term (ΔG_SA = −7.4 ± 0.8 kcal mol⁻¹) further contributed to binding through hydrophobic effect.

**Figure 6:**
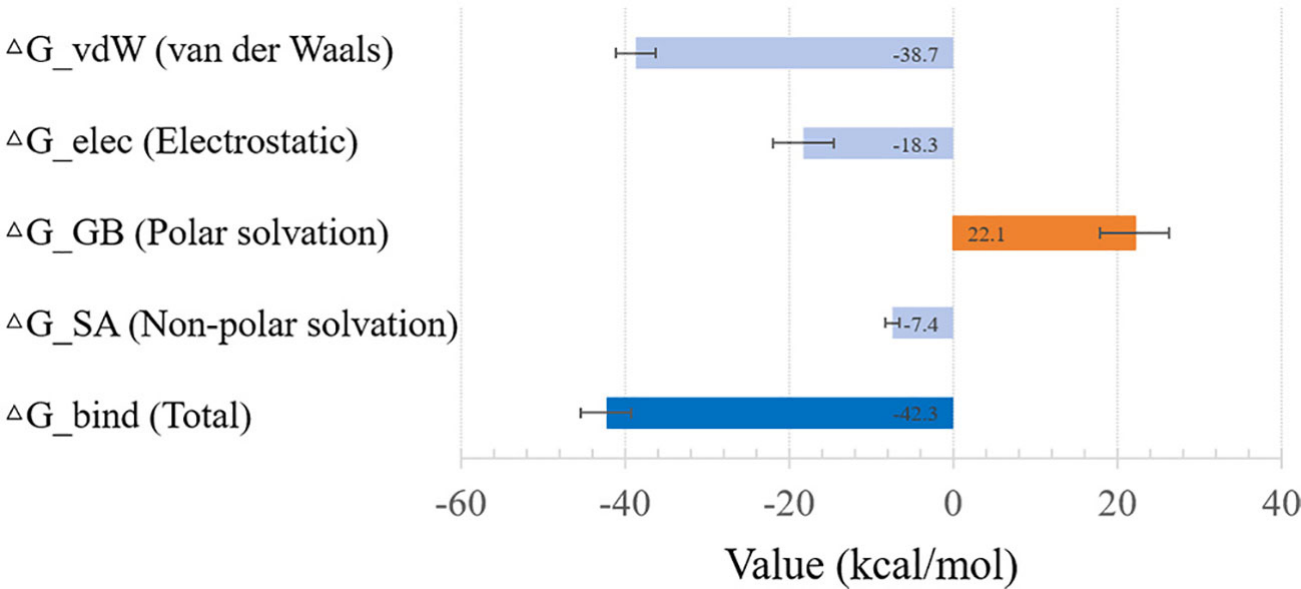
MM-GBSA binding free energy components for the DLL3–IMP-3 complex. Based on this quantitative decomposition, we propose the following systematic structure-activity relationship (SAR) strategies for future optimization: (i) Conservative substitution of Trp² with 5-fluoro-tryptophan or 1-methyl-tryptophan to probe the steric and electronic tolerance of the deep hydrophobic pocket; (ii) Leu^4^ → norleucine (Nle) or cyclohexylalanine (Cha) substitution to evaluate distance and steric constraints; (iii) Leu^6^ → tert-leucine (Tle) substitution to increase side-chain branching and restrict conformational flexibility. For stability improvement: (iv) backbone N-methylation at non-pharmacophoric positions (Ser¹ or Ser^5^;); and (v) D-amino acid scanning at Pro^7^ and Ser^5^; to evaluate stereochemical effects on plasma stability. For pharmacokinetic optimization: (vi) PEGylation or fatty acid acylation at Pro^7^ to modulate plasma half-life; and (vii) alternative cyclization strategies to further enhance *in vivo* stability. These systematic SAR approaches, guided by the per-residue energy decomposition presented here, provide a rational framework for second-generation IMP-3 development.

Per-residue free energy decomposition identified three IMP-3 residues as the primary pharmacophoric hotspots: Trp² (contribution: −9.8 kcal mol⁻¹), Leu^4^ (−7.3 kcal mol⁻¹), and Leu^6^ (−6.9 kcal mol⁻¹). His³ and Ser¹ contributed −4.2 and −3.6 kcal mol⁻¹ respectively, primarily through electrostatic and hydrogen-bonding terms. Pro^7^ contributed minimally to direct binding energy (−1.1 kcal mol⁻¹) but plays an essential structural role in enforcing the β-turn geometry that pre-organizes the pharmacophoric residues. These data provide a quantitative, residue-level roadmap for IMP-3 structure-activity relationship (SAR) optimization: Trp², Leu^4^, and Leu^6^ are critical binding determinants to be preserved or conservatively substituted, while Pro^7^ and the Ser positions offer opportunities for pharmacokinetic modulation without sacrificing core binding affinity. This is consistent with the conclusion of the affinity experiment in **Figure 6**.

## 4 Discussion

The present study employed an integrated AI-driven multi-omics pipeline to systematically identify DLL3 as a high-priority therapeutic target in GBM and to develop IMP-3 as its corresponding high-affinity cyclic peptide carrier for RDC applications.

Differential expression analysis identified 2,969 significantly dysregulated genes in GBM versus normal brain tissue, of which 1,343 were upregulated and 1,626 were downregulated. Hierarchical clustering confirmed distinct transcriptional separation between GBM and normal cohorts, with GBM-enriched genes including inflammation-related (C1QA, C1QC, TREM2), immune regulatory (HLA-DRA, HLA-DRB1), and extracellular matrix remodeling genes (SERPINE1, TNC), collectively reflecting the aggressive neuroinflammatory and invasive biology characteristic of GBM (7, 8). GO and KEGG enrichment analyses revealed predominant dysregulation of synaptic signaling, ion channel activity, and neuroimmune pathways, consistent with the established co-option of neuronal circuitry by GBM cells to sustain proliferative and invasive programs. These enrichment findings contextualize DLL3 within a broader oncogenic signaling landscape in which Notch pathway dysregulation intersects with synaptic remodeling and CSC maintenance to drive tumor aggressiveness.

The machine learning pipeline progressively refined 2,969 DEGs to a single prioritized target through three sequential dimensionality-reduction steps. Membrane protein annotation and Spearman correlation screening reduced 202 candidates to 15 non-redundant genes; LASSO regression with ten-fold cross-validation further selected seven features (DLL3, HAS2, MRC2, F2R, IL13RA2, CNGA3, CHRNA9) at the optimal λ minimizing binomial deviance. The resulting XGBoost classifier achieved a cross-validated AUC-ROC of 0.961, confirming the collective discriminative power of the selected feature set. SHAP decomposition identified DLL3 as the dominant predictive feature: it exhibited the highest mean absolute SHAP value, a monotonically positive expression-to-SHAP relationship exclusive to GBM samples, and near-complete distributional separation from normal brain controls with no distributional overlap. The combined SHAP contribution of the top three features (DLL3, HAS2, MRC2) exceeded 80% of total model prediction weight, further underscoring the biological centrality of DLL3. These multi-layered computational findings were further supported by DLL3’s superior GBM-specific overexpression (log_2_FC = 2.65), restricted normal-tissue expression profile minimizing off-target risk, established clinical translatability through rovalpituzumab tesirine (34), and availability of a structurally tractable extracellular domain (UniProt: Q9NYJ7) for rational peptide design.

Several DLL3-targeting agents have been reported in the literature, most notably rovalpituzumab tesirine (Rova-T), an antibody-drug conjugate (ADC) comprising a humanized anti-DLL3 monoclonal antibody conjugated to a pyrrolobenzodiazepine dimer toxin. Rova-T demonstrated promising preclinical activity in DLL3-positive small-cell lung cancer (SCLC) and advanced to phase III clinical evaluation (NCT03033511, NCT03061812), although clinical development was subsequently discontinued due to limited efficacy and off-target toxicity (34, 36, 37). More recently, bispecific T-cell engagers targeting DLL3 (e.g., AMG 757, tarlatamab); and DLL3-directed chimeric antigen receptor (CAR) T cells have entered clinical investigation for neuroendocrine tumors (38). Compared to these macromolecular biologics (~150 kDa antibodies or ~55 kDa bispecific engagers), IMP-3 offers several distinct advantages for radionuclide-based theranostic applications: (i) its small molecular size (~0.8 kDa) facilitates deeper tumor tissue penetration and potentially improved tumor-to-background contrast for imaging; (ii) the cyclic peptide scaffold provides enhanced proteolytic stability compared to linear peptides; (iii) the well-defined conjugation site (Pro^7^) enables site-specific bifunctional chelator attachment without compromising binding affinity; and (iv) the short plasma half-life characteristic of peptides is advantageous for radionuclide-based approaches, as it minimizes prolonged radiation exposure to normal tissues. To our knowledge, IMP-3 represents the first cyclic peptide specifically designed to target DLL3 for radionuclide-based theranostic applications, underscoring its novelty and translational potential.

Recently, Ding and Yeong comprehensively reviewed the evolving landscape of DLL3-targeted therapies across multiple modalities, including ADCs, BiTEs, CAR-T cell therapy, near-infrared photoimmunotherapy, and radiopharmaceutical therapy, highlighting both clinical advancements and the challenges encountered in translating these approaches into practice (39).

An important consideration for GBM-targeted theranostic development is the blood-brain barrier (BBB) penetration characteristics of the peptide carrier. For systemically administered radionuclide-based conjugates, the BBB represents a major obstacle. However, it is well established that GBM disrupts the BBB locally, particularly in contrast-enhancing tumor regions where the barrier is compromised, enabling macromolecular and peptide-based agents to extravasate via a passive enhanced permeability and retention (EPR)-like effect (40). Cyclic peptides of molecular weight < 2 kDa, such as IMP-3 (~0.8 kDa), are generally considered favorable for brain tumor delivery compared to full antibodies (~150 kDa). The physicochemical properties of IMP-3-including its compact cyclic structure, moderate hydrophilicity, and net neutral charge at physiological pH-are compatible with passive diffusion across disrupted BBB regions. Moreover, small cyclic peptides can potentially exploit receptor-mediated transcytosis mechanisms when conjugated to appropriate BBB shuttle peptides or ligands. The planned DOTA-IMP-3 conjugate for ¹^77^Lu or ²²^5^;Ac radiolabeling will retain favorable molecular weight characteristics. Future *in vivo* studies using orthotopic GBM xenograft models will directly evaluate IMP-3 conjugate brain tumor accumulation, intratumoral distribution, and the impact of BBB integrity on delivery efficiency. Recent advances in peptide-conjugated lipid nanoparticles for siRNA delivery across the BBB further suggest opportunities for optimizing IMP-3 delivery through conjugation with BBB-penetrating moieties (26).

Integrative proteomic profiling has further identified PARP1 as a highly expressed therapeutic target in SCLC, demonstrating that PARP inhibition suppresses tumor growth and enhances chemotherapy efficacy, providing a precedent for proteomics-driven target discovery in aggressive neuroendocrine tumors (41).More recently, Rudin et al. comprehensively reviewed the clinical development of DLL3-targeted therapies, including the lessons learned from Rova-T, the regulatory approval of tarlatamab as the first DLL3-targeted bispecific T-cell engager, and the emerging pipeline of BI 764532, HPN328, and DLL3-directed CAR-T cell therapies, providing an up-to-date framework for understanding the rapidly evolving DLL3 therapeutic landscape (37).

Cell-based validation by confocal microscopy and Western blot confirmed DLL3 protein expression across U87, U251, and T98G glioma cell lines, with highest expression in U87 cells, providing essential protein-level corroboration of the transcriptomic prediction and confirming plasma membrane accessibility of the DLL3 extracellular domain for circulating RDC conjugates. Molecular docking and 100-ns all-atom MD simulation of five candidate peptides identified IMP-3 (cyclo-[Ser-Trp-His-Leu-Ser-Leu-Pro]) as the lead compound by a substantial margin in binding free energy (MM-GBSA ΔG_bind = −42.3 ± 3.1 kcal mol⁻¹), dominated by van der Waals contributions (ΔG_vdW = −38.7 ± 2.4 kcal mol⁻¹) consistent with deep hydrophobic burial within the DLL3 EGF3-EGF4 binding groove. Per-residue energy decomposition identified Trp² (−9.8 kcal mol⁻¹), Leu^4^ (−7.3 kcal mol⁻¹), and Leu^6^ (−6.9 kcal mol⁻¹) as the dominant pharmacophoric hotspots, providing a quantitative SAR roadmap for future affinity optimization while identifying Pro^7^ as the optimal conjugation site for bifunctional chelator attachment without pharmacophoric disruption. *In vitro* flow cytometry and confocal fluorescence imaging confirmed specific, receptor-mediated IMP-3 binding to DLL3-positive cells, with a 9.3-fold mean fluorescence intensity differential over DLL3-negative controls (p < 0.001) and 87.4% competitive displacement by unlabeled IMP-3, in full concordance with the computational binding predictions. Membrane co-localization of the fluorescent IMP-3 probe with DLL3 on glioma cell surfaces further validated target engagement at the cellular level, confirming IMP-3 as a functionally verified DLL3-targeting scaffold suitable for ^225^Ac or ¹^77^Lu chelator conjugation in subsequent RDC development. (Cohen’s d = 9.82, 95% CI: 2176–2926, p < 0.001).

It must be explicitly stated that DLL3 is employed in the present study as a tumor-selective surface delivery antigen for RDC targeting, not as a validated causal oncogenic driver. The therapeutic rationale for RDC targeting requires: (i) selective tumor surface overexpression, (ii) near-absent normal tissue expression, (iii) extracellular structural accessibility, and (iv) a validated therapeutic window-DLL3 satisfies all four conditions. Whether DLL3 is mechanistically necessary or sufficient for GBM initiation and maintenance is a separate and important biological question that requires dedicated functional genomics studies and is beyond the scope of the present work.

Limitations of the present study include the use of a computationally predicted rather than experimentally determined DLL3 structural model, the restriction of cell-based validation to established GBM cell lines rather than primary patient-derived neurosphere cultures. Progression to patient-derived glioma stem cell (PDGSC) models and primary tumor organoids represents the natural and prioritized next translational step following identification of a validated hit scaffold and the absence of *in vivo* biodistribution and efficacy data for radiolabeled IMP-3 conjugates. Future studies will address these gaps through cryo-EM structural determination of the DLL3-IMP-3 complex, radiolabeling of DOTA-IMP-3 with ¹^77^Lu and ²²^5^;Ac, and orthotopic GBM xenograft biodistribution and therapeutic efficacy evaluation. We also acknowledge that DLL3 protein expression validation in a larger cohort of human GBM tissues using immunohistochemistry (IHC) on tissue microarrays (TMAs) would further strengthen the translational significance. Future studies will incorporate systematic IHC analysis of DLL3 expression in surgically resected GBM specimens and multi-region TMAs to evaluate intratumoral heterogeneity and correlate DLL3 expression with clinicopathological parameters.

## 5 Conclusions

This study presents a fully integrated, AI-driven computational and experimental pipeline for GBM-specific cell surface target identification and rational peptide carrier design for radionuclide therapy. Through the sequential application of multi-omics data integration, pairwise gene correlation-based feature deduplication, LASSO regression, SHAP-guided XGBoost explainability analysis, AlphaFold2 structural prediction, Glide XP molecular docking, and 100-ns GROMACS all-atom MD simulation with MM-PBSA binding free energy estimation, we identify DLL3 as the highest-priority GBM-specific plasma membrane target and develop IMP-3 (cyclo-[Ser-Trp-His-Leu-Ser-Leu-Pro]) as its first-generation cyclic peptide carrier. External independent validation in the CGGA cohort (AUC = 0.783) confirms DLL3’s generalizability as a GBM biomarker and model target. *In vitro* fluorescence imaging demonstrates a 9.3-fold target-selective MFI differential and 87.4% competitive displacement, confirming IMP-3 as an experimentally validated DLL3-binding scaffold. The model achieved an AUC of 0.783 (95% CI: 0.741–0.825), with sensitivity of 0.742, specificity of 0.801, accuracy of 0.775, and positive predictive value of 0.763. Calibration analysis demonstrated good agreement between predicted and observed probabilities (Brier score = 0.154; **Supplementary Figure 4**), further supporting model generalizability. External validation yielded an AUC of 0.783 (95% CI: 0.741–0.825) with sensitivity of 0.742 and specificity of 0.801, confirming DLL3’s robust generalizability as a GBM biomarker. *In vitro* flow cytometry demonstrated a 9.3-fold target-selective MFI differential (p < 0.001, Cohen’s d = 9.82) and 87.4% competitive displacement (p < 0.001, paired t-test, n = 3), confirming IMP-3 as an experimentally validated DLL3-binding scaffold for radionuclide-based theranostic development.

Prioritized future work includes DOTA-IMP-3 radiolabeling with ¹^77^Lu and ²²^5^;Ac; *in vitro* internalization and cytotoxicity assays; orthotopic GBM xenograft biodistribution and therapeutic efficacy evaluation; and immunohistochemical DLL3 expression profiling in human GBM tissue microarrays.

## Data Availability

The datasets presented in this study can be found in online repositories. The names of the repository/repositories and accession number(s) can be found below: https://xenabrowser.net, UCSC https://gtexportal.org, GTEx.
