# Supplementary material for: Machine learning-driven identification of DLL3 as a molecular target and development of a DLL3-binding cyclic peptide for glioblastoma
Source: Front Oncol. 2026 Jul 14;16:1847670. doi: 10.3389/fonc.2026.1847670 (PMC13407090; doi:10.3389/fonc.2026.1847670)

Supplementary Material

# Supplementary Data

# Supplementary Figures and Tables

## Supplementary Figures


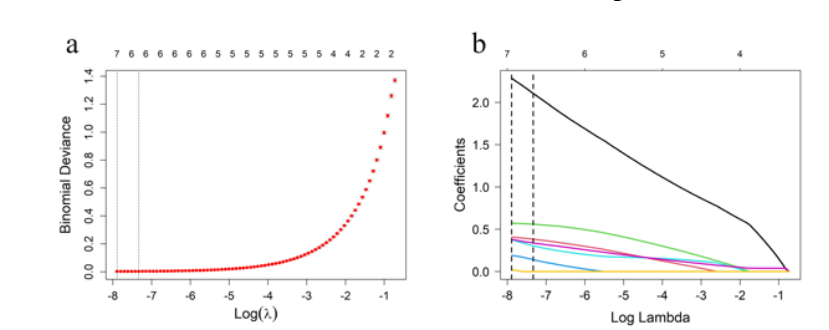


Supplementary Fig.1. Machine learning-driven prioritization and validation of DLL3 as a therapeutic target in GBM. (a) LASSO regression: Binomial deviance (left axis) and coefficient retention (right axis) across log(λ) values. Optimal λ (dashed vertical line) minimizes deviance, selecting seven genes (diamond markers). (b) Coefficient trajectories of candidate genes; lines shrink to zero as λ increases.


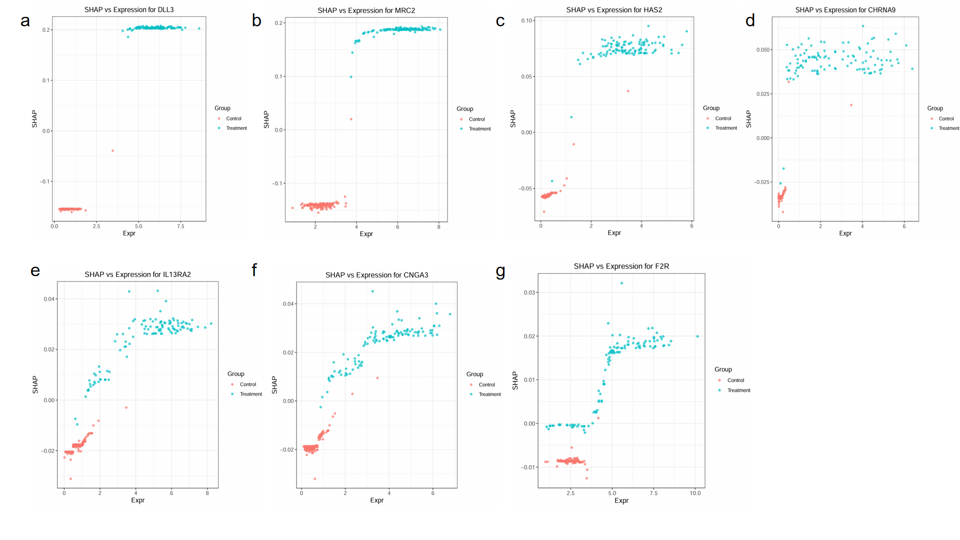


Supplyment Fig. . Scatter plots of the association between expression levels and SHAP values for seven genes, with the horizontal axis representing the expression levels (Expr) of the seven different genes and the vertical axis representing the SHAP values of the corresponding genes; red dots represent control group samples and cyan dots represent treatment group samples, illustrating the distribution patterns of the contribution of gene expression levels to model prediction (SHAP values) in different groups.


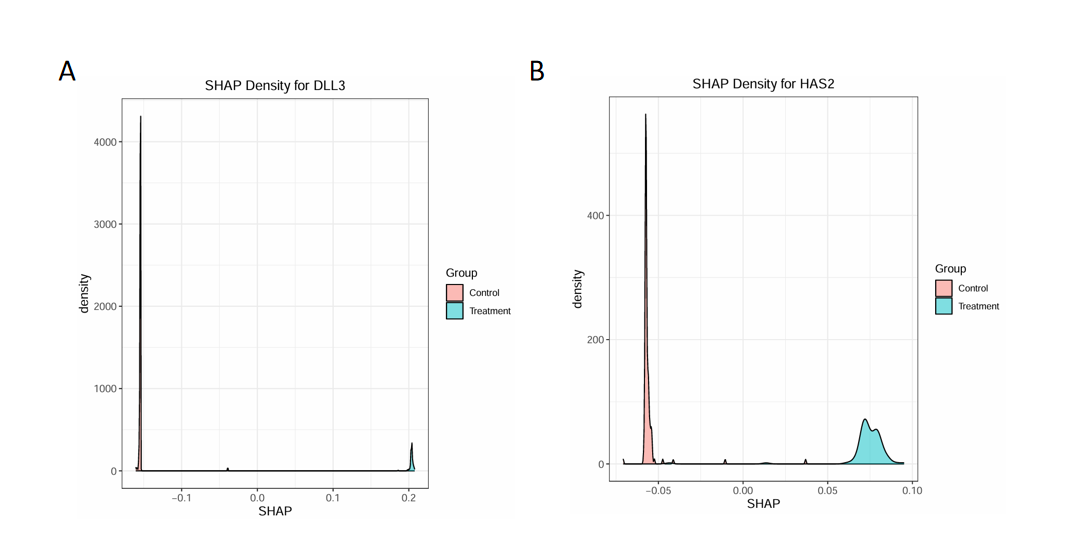


Supplement Fig.3. Density distribution plots of SHAP values for the DLL3 and HAS2 genes. (A) The horizontal axis represents the SHAP values of the gene, and the vertical axis represents density; the red area denotes samples from the control group, and the cyan area denotes samples from the disease
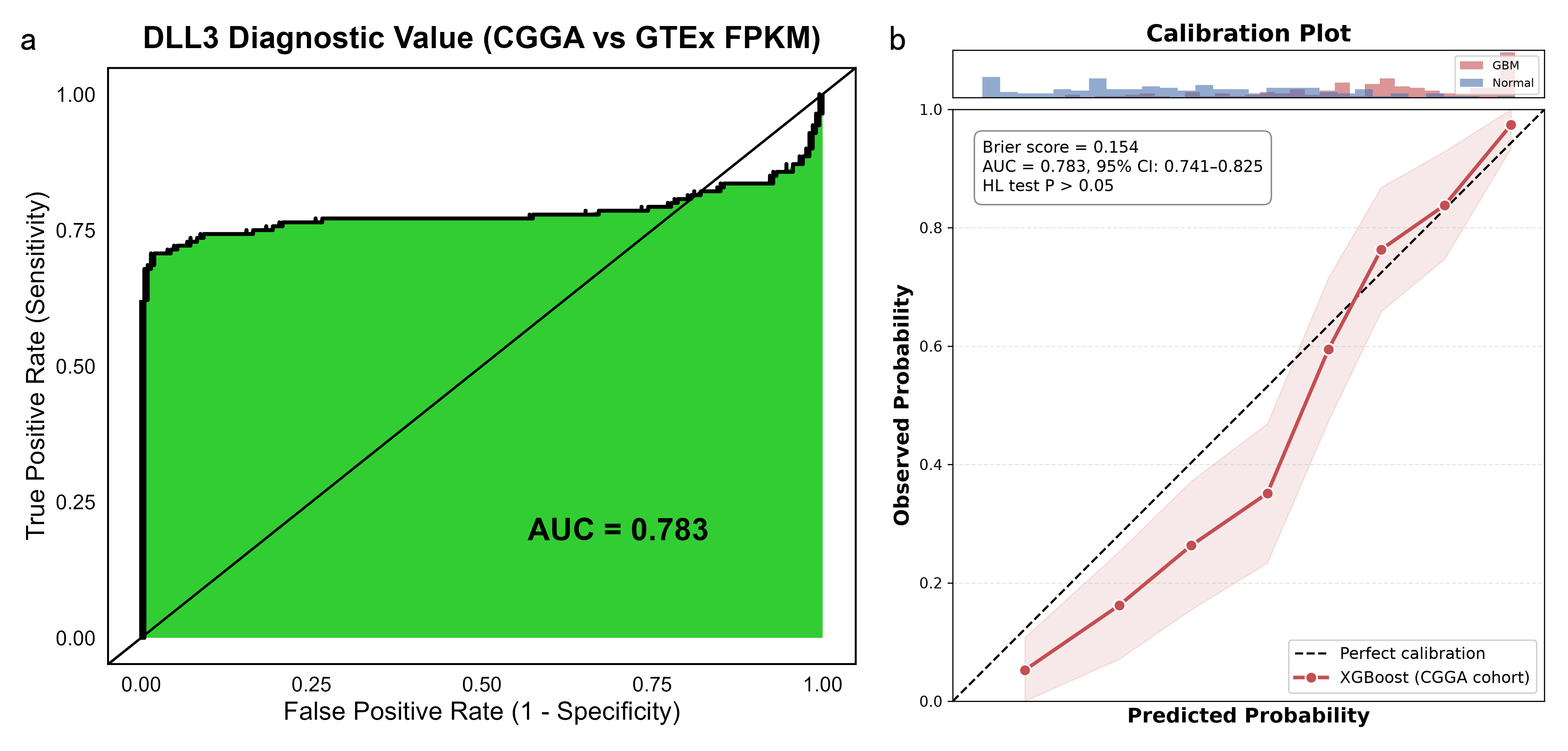
group, illustrating the distribution density characteristics of SHAP values of the HAS2 gene in the two groups of samples.

Supplementary Figure 4. External validation of the XGBoost model in the CGGA cohort. (a) ROC curve analysis showing AUC = 0.783 (95% CI: 0.741–0.825). (b) Calibration plot demonstrating agreement between predicted and observed probabilities (Brier score = 0.154).

Supplement Table 1. MM-GBSA binding free energy components for the DLL3–IMP-3 complex


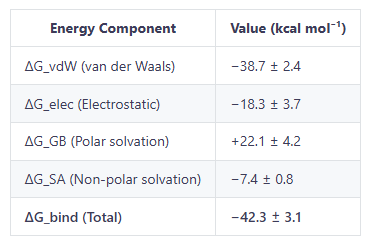


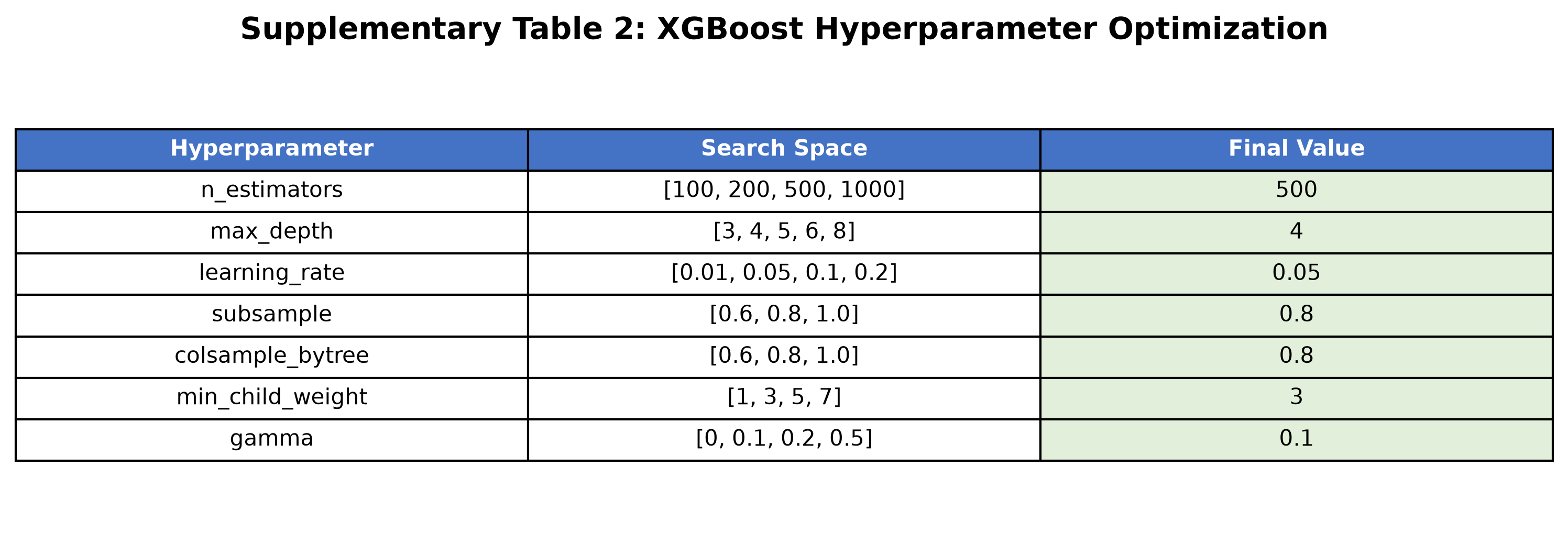

Supplement: Supplementary file 1 [file DataSheet1.docx]
